# Near-chromosomal-level genome of the red palm weevil (*Rhynchophorus ferrugineus*), a potential resource for genome-based pest control

**DOI:** 10.1038/s41597-024-02910-3

**Published:** 2024-01-06

**Authors:** Naganeeswaran Sudalaimuthuasari, Biduth Kundu, Khaled M. Hazzouri, Khaled M. A. Amiri

**Affiliations:** 1 Khalifa Center for Genetic Engineering and Biotechnology, United Arab Emirates University, Al Ain, UAE; 2 Department of Biology, College of Science, United Arab Emirates University, Al Ain, UAE

**Keywords:** Computational biology and bioinformatics, Genetics

## Abstract

The red palm weevil (RPW) is a highly destructive pest that mainly affects palms, particularly date palms (*Phoenix dactylifera*), in the Arabian Gulf region. In this study, we present a near-chromosomal-level genome assembly of the RPW using a combination of PacBio HiFi and Dovetail Omini-C reads. The final genome assembly is around 779 Mb in size, with an N50 of ~43 Mb, consistent with our previous flow cytometry estimates. The completeness of the genome was confirmed through BUSCO analysis, which indicates the presence of 99.5% of BUSCO single copy orthologous genes. The genome annotation identified a total of 29,666 protein-coding, 1,091 tRNA and 543 rRNA genes. Overall, the proposed genome assembly is significantly superior to existing assemblies in terms of contiguity, integrity, and genome completeness.

## Background & Summary

The RPW (*Rhynchophorus ferrugineus* (Olivier)) is an extremely destructive pest that poses a significant threat to palm trees (Arecaceae) in various agroecosystems^1,2^. Belonging to the family Curculionidae, this Coleopteran pest beetle is native to Southeast Asia^3^. Over the past three decades, RPW has spread extensively, reaching the Arabian Gulf, Mediterranean regions, and other parts of the world^1,3,4^. In the Arabian Gulf, the RPW inflicts severe damage on *Phoenix dactylifera* L. (date palm), which is a crucial and important economic crop. Each year, a large number of date palms are affected by RPW, resulting in multimillion-dollar losses in yield^4^. Currently, traditional agricultural practices are employed to manage RPW infections, including the surveillance of plants for RPW infestation, capturing weevils, treating infected plants with pesticides, and finally removing/isolating infected plants from healthy plants^5^.

Due to recent advances in sequencing technology, genomics-based insect management has been suggested^6–9^. Initially, transcriptome studies were carried out in RPW to identify specific genes mainly expressed during its development and infection of palm trees^10^. However, the lack of a complete genome assembly has made genome-based management difficult. To tackle this problem, we published a first draft genome (rfM v1) of RPW with the aim of identifying important gene families relevant to the destructive life history trait of this species^11^. In this initial assembly (rfM v1), we used 10X, Illumina and Nanopore sequencing technologies; however, the lack of proper genetic map forced us to use the *Tribolium castaneum* genome for synteny-based pseudochromosome generation. Given the lack of a genetic map as well as genome-wide chromatin interaction information (such as Hi-C), our previous assembly had mis-ordered and mis-oriented scaffolds, which resulted in an apparent higher gene duplication level. In 2021, Guilherme *et al*.^12^ published a second draft haplotype-resolved RPW genome assembly. Recently, a third draft genome assembly was also deposited in public repository^13^ (10.5281/zenodo.6878576). The second and third genomes assembly reported a genome size of ~550 Mb and ~1.16 GB respectively and the contiguity and completeness of these genomes remain problematic and the assembly is still at the contig/scaffold level.

**Fig. 1:**
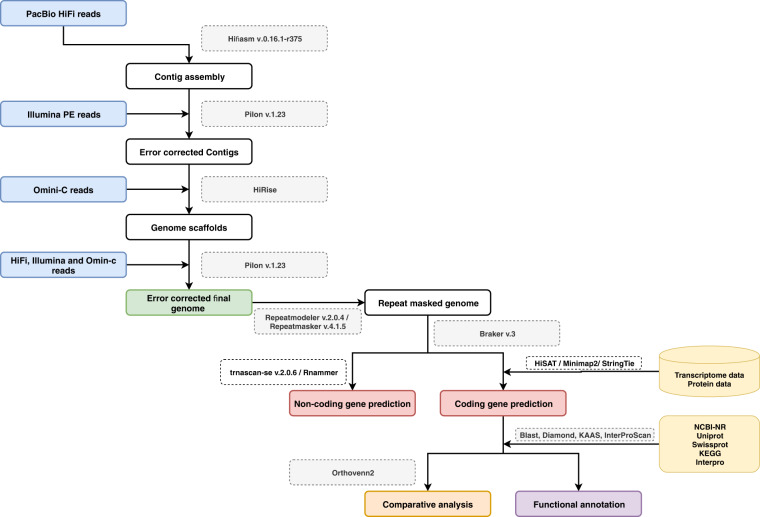
Detailed workflow pipeline for *de novo* whole-genome assembly and annotation of *Rhynchophorus ferrugineus*.

To improve genome contiguity and completeness of the RPW reference genome, a chromosomal-level reference assembly is required. Such a chromosome assembly will provide an important and complete resource to study RPW diversity, genomic variation, molecular evolution, and environmental adaptation, which could eventually lead to molecular genetic-based pest control^7,8,14^. In this study, we report a near-chromosomal-level genome assembly of RPW^15^, achieved using both PacBio HiFi and Illumina based Omini-C data^16^. The full genome assembly and annotation workflow is shown in Fig. 1.

## Methods

### Sample collection, DNA isolation and genome sequencing

An adult RPW male (marked as W39M) was collected from a date palm farm (Al Foah farm) located at Al Ain, UAE. Prior to DNA isolation, the sample underwent thorough surface cleaning and was subsequently flash-frozen with liquid nitrogen. High molecular weight genomic DNA isolation was carried out on Maxwell® RSC 48 (Promega Corporation, Wisconsin, USA) using Maxwell® RSC Tissue DNA kit (Promega Corporation, Wisconsin, USA). In total, ~15.5 micrograms (~310 ng/microL) of DNA were extracted from the W39M sample and used for the preparation of the whole genome sequencing (WGS) library.

A PacBio WGS Hifi library (CCS method, ~10–20 kb insert size) was constructed using the SMRT bell TM Template kit (version 1.0) according to manufacturer’s protocol. During library preparation, BluePippin System (Sage Science, MA, USA) was used for library size selection and library quality was confirmed on a Qubit® 2.0 Fluorometer (Thermo Fisher Scientific™, Waltham, MA, USA). Finally, library insert size was assured by Bioanalyzer (Agilent 2100, Agilent Technologies) and sequenced on the PacBio Sequel II platform. The sub reads generated from the PacBio Sequel II platform were converted into HiFi reads using CCS v.4.20 tool (https://github.com/PacificBiosciences/ccs/releases/tag/v4.2.0).

The Dovetail proximity ligation Omni-C library was prepared using Dovetail® Omni-C® Kit (USA) according to manufacturer’s instructions. During library preparation, chromatin structure of the RPW sample was fixed using formaldehyde and DNA extraction carried out. DNA was digested with DNAse I restriction enzyme followed by proximity ligation. Finally, a Illumina PE sequencing compatible library was generated from fragmented DNA using NEBNext Ultra kit. The library was then sequenced on the Illumina HiSeqX system at the Dovetail facility (USA). For genome error correction and gene annotation, we have used currently generated (HiFi and Omini-C^16^), previously generated (Ilumina WGS and transcriptome^11^) and publicly available (Pacbio Isoseq, transcriptome and proteome) data^12,17,18^.

### Genome size estimation, genome assembly and scaffolding

**Fig. 2:**
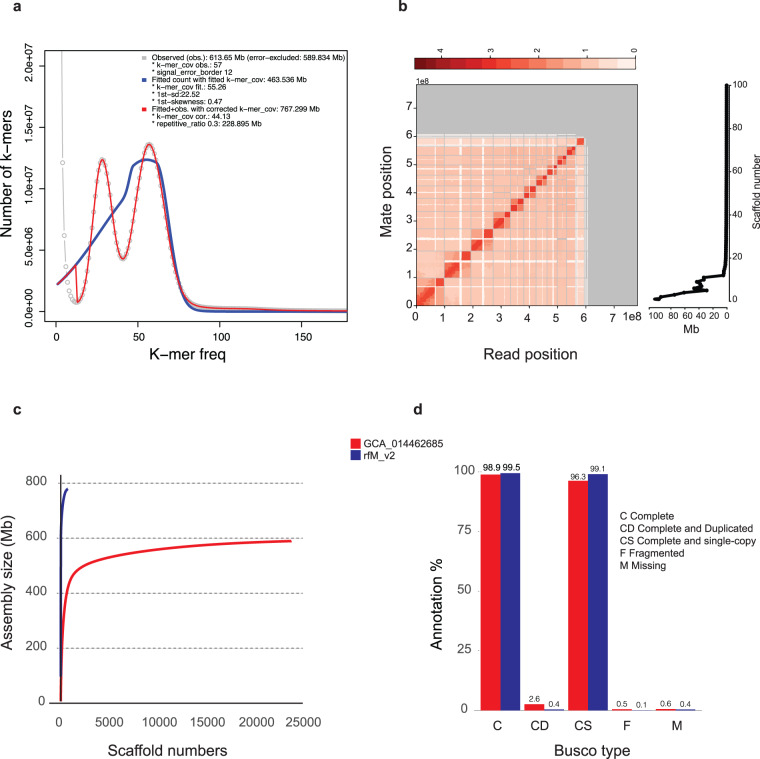
Genome assembly assessment and comparison. (**a**) The histogram generated by findGSE with a k-mer size of 21 is displayed below. The observed k-mer frequency is depicted by the gray line, while the teal line represents the fitted model for the heterozygous k-mer peak. The blue line represents the fitted model without k-mer correction, and finally, the red line represents the fitted model with k-mer correction, which is utilized to estimate the size of the genome. (**b**) A left panel with a Hi-C contact map is used to represent the genome assembly, showing the proximity of genomic regions in three-dimensional space as a contiguous linear arrangement. Each cell in the contact map represents sequencing data that confirms the linkage between two specific regions. Gray lines are used to separate scaffolds, and the density of the map indicates the degree of fragmentation, with higher density indicating more fragmentation. The right panel, depict a plot showing the size distribution in Mega base (Mb) of each scaffold in the genome. (**c**) This plot compares the rfMv2 genome in this study to a published genome (GCA_014462685) of RPW by plotting the cumulative sequence length (y-axis) against the increasing number of scaffolds (x-axis). (**d**) The comparison between the rfMv2 genome presented in this study and the published genome (GCA_014462685) was visualized using grouped bar charts. These charts depict the BUSCO analyses for the insecta_odb10 gene sets. The height of the bars represents the percentage of genes found in each assembly relative to the total gene set. Additionally, x axis of each of the grouped bar charts are labeled with the Initials based on the BUSCO status: M for missing genes, F for fragmented genes, CD indicates complete and duplicated genes, and CS represents complete and single-copy genes.

The bam file (HiFi data) generated from PacBio Seqel II was initially converted into fastq file using Bam2Fastq v.1.1 program (https://github.com/jts/bam2fastq). In total, ~2.1 million HiFi long reads were generated for this study. The average length of the reads was around 17.2 Kb, with an N50 value of 17,221 bp. The total size of the generated reads amounted to ~ 37 Gb, providing a sequencing coverage of ~51X (coverage was calculated based on the flow cytometry value from our previous study^11^). Prior to the genome assembly, we estimated genome size of RPW using HiFi long reads and Illumina data (male data; data from our previous study^11^) by kmerfreq v.1^19^ and gce v.1.0.2 (https://github.com/fanagislab/GCE) software. Based on the size estimation analysis, the estimated genome size of RPW was determined to be ~ 603 Mb. To obtain a more accurate estimation of the theoretical genome size, we employed several genome size estimation tools, including Jellyfish v.2.3^20^, GenomeScope v.1^21^, GenomeScope v. 2^22^, and R program-based approaches including findGSE^23^. Interestingly, the results from these approaches varied within a range from ~499 Mb to ~767.229 Mb (Fig. 2a). The observed discrepancies in the estimated genome size could potentially be attributed to the heterozygosity and complex organization of the RPW genome^12,14^ (Supplementary Fig. 1a and 1b).

The genome assembly was carried out using Hifiasm v.0.16.1 software^24^ with default parameters. The genome assembly resulted in 750 contigs with an assembly size of 784 Mb (N50: 20.2 Mb and G + C%: 33.6) (Table 1). The assembled contigs were error corrected by Pilon v.1.23^25^ using Illumina data. Furthermore, contigs were scaffolded and pseudochromosomes were generated using Omni-C data. In total, ~115 million Omni-C reads (~44X) were generated for this study. For scaffolding, we used four different scaffolding programs (Supplementary Table 1); among them HiRiSE v.X^26^ generated better scaffolds with a size of ~784 Mb (N50: ~43.4 Mb, G + C%: 33.6 and longest scaffold length: ~97.8 Mb) (Table 1). HiRise generated 37 scaffolds with the size > 1 Mb; among them, 11 scaffolds which sizes are more than 29 MB, which are considered as pseudochromosomes. Generated scaffolds were error corrected using Illumina, HiFi and Omni-C data, which resulted in a final genome size of 779 Mb (N50: ~43 Mb, G + C%: 33.6 and longest scaffold length: ~97.2 Mb) with 11 chromosome level scaffolds (rfM v2) (Table 1) (Fig. 2b, Supplementary Fig. 2). The final assembled genome size is ~2 -~36% higher than the estimated genome size; the discrepancy between the estimated genome size and assembled genome size happened due to the complexity and heterozygosity nature of the RPW genome^12,14^. But the assembled genome size is almost same size of the previously reported genome size^11^. Moreover, the total genome size is ~7% higher than the flow cytometry-based estimated genome size^11^. We observe a huge improvement in terms of completeness and contiguity plotting the number of scaffolds that represents the maximum genome size in term of completeness and contiguity between our sequenced genome rfM2 and NCBI GCA_014462685 weevil genome (Fig. 2c). We carried out BUSCO v.4.1.4 (insecta_odb10)^27^ analysis on assembled contigs, scaffolds and the final genome and compared that to the GCA_014462685 genome (Fig. 2d, Table 1). In total, 99.5% BUSCO universal single-copy orthologous genes were annotated from the final assembly. Moreover, duplicated and missing BUSCO genes percentage was estimated as 0.4%, which were lesser than the previously released genome assembly (rfM v1)^11^ as well as GCA_014462685 genome^12^ (Fig. 2d).

**Table 1:** Genome assembly and annotation statistics.

	Contigs	Scaffolds	Final genome
No. of sequences	750	728	721
Genome size (Mb)	~784	~784	~779
Number of Pseudochromosomes		11	11
G + C %	~33.6	~33.6	~33.6
N %	0	0.00029	0.00018
Max length (Mb)	~56.6	~97.8	~97.2
N50 length (bp)	20287965	43484440	43002243
*BUSCO (n: 1367)	C:99.4% [S:99.0%, D:0.4%] F:0.2% M:0.4%	C:99.5% [S:99.1%, D:0.4%] F:0.1% M:0.4%	C:99.5% [S:99.1%, D:0.4%] F:0.1% M:0.4%
Protein coding genes			29666
Total mRNAs			32652
Total exons			149788
Total introns			117160
NR annotation			29747 (~91.1%)
UniProt annotation			29755 (~91.1%)
SwissProt annotation			14823 (~45.3%)
InterPro annotation			18302 (~56.0%)
KEGG annotation			9930 (~30. 4%)

^*^BUSCO; C: Complete BUSCOs, S: Complete and single-copy BUSCOs, D: Complete and duplicated BUSCOs, F: Fragmented BUSCOs, M: Missing BUSCOs and n: Total BUSCO groups.

**Fig. 3:**
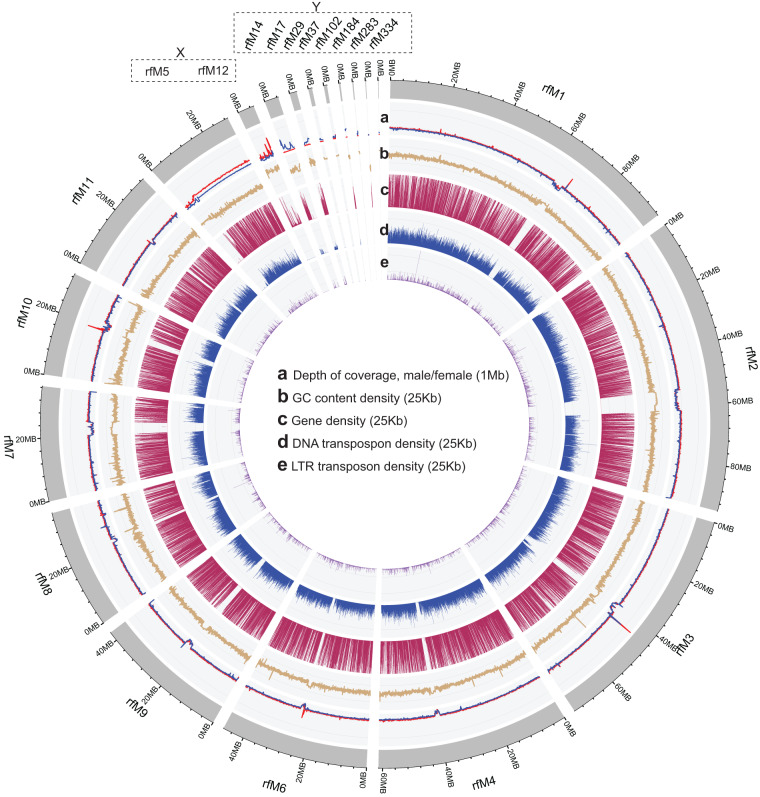
Distribution of depth of coverage and genome features. Circos plot depicting genome features across the 11 RPW chromosomes, highlighting with dotted squares the X and Y chromosomes. (**a**) RPW chromosomes (rsfM1-rsfM11-X, Y) depth of coverage in male and female (**b**) GC content percentage. (**c**) Gene density across the genome. (**d**) Distribution of repeat elements: DNA transposons (blue bar), (**e**) retrotransposon TEs (red bar). For tracks (**b**–**e**), a window size of 25Kb was used, whereas for tracks (**a**), the size was increased to 1 Mb.

Based on previous karyotypic studies, the chromosome number of male RPW has been estimated as 2n = 22 (10 + XY)^28,29^. The Y chromosome is smaller in size compared to the other chromosomes^29^. To identify X and Y chromosome from the final assembly, we aligned the male and female RPW Illumina short reads to the final assembly using bwa v.0.7.17 alignment tool^30^ and calculated the alignment coverage (vertical and horizontal) using Mosdepth v.0.3.3 tool^31^. The 10 autosomes (A) have almost similar coverage and depth in both male and female Illumina data. Based on the reads, horizonal coverage and high vertical depth of the female Illumina data, we determined pseudochromosome rfM5 (ASCQM010000005; size ~29 Mb) and scaffold rfM12 (JASCQM010000012; size: ~5.2 Mb) are part of X chromosome. Similarly, based on the higher read coverage and high vertical depth of the male Illumina data, scaffolds rfM14 (JASCQM010000014), rfM17(JASCQM010000017), rfM29 (JASCQM010000029), rfM37 (JASCQM010000037), rfM102 (JASCQM010000102), rfM184 (JASCQM010000184), rfM283 (JASCQM010000283), rfM334 (JASCQM010000334) which amounting the total size of ~10 Mb, are identified as a part of Y chromosome (Fig. 3, Supplementary Fig. 3).

### Repeat finding, gene prediction and functional annotation

The repeat regions found in the genome were identified and masked through RepeatModeler v. 2.0.4^32^ and RepeatMasker v. 4.1.5^33^ tools. The *de novo* repeat library from the genome was constructed using RepeatModeler (-database weevil_rdb -threads 80 -LTRStruct). From the repeat library, possible protein/transcripts related sequence were removed, and RPW v2 genome was masked using RepeatMasker (-e ncbi -pa 80 -norna -lib -a -xsmall -gff). In total, ~31% (~240 Mb) of genome was identified as repeats. Similar to Hymenoptera, Coleoptera RPW DNA transposons are more prevalent in the genome compared to long terminal repeats (LTRs)^34^ (Fig. 3). Interestingly, we observe a GC bias for one scaffold, X chromosome (rfM12) (Fig. 3), similar to palindromes hotspots in the human X chromosome, which is consistent with GC-biased gene conversion during recombination, as opposed to recombination suppression^35^.

**Fig. 4:**
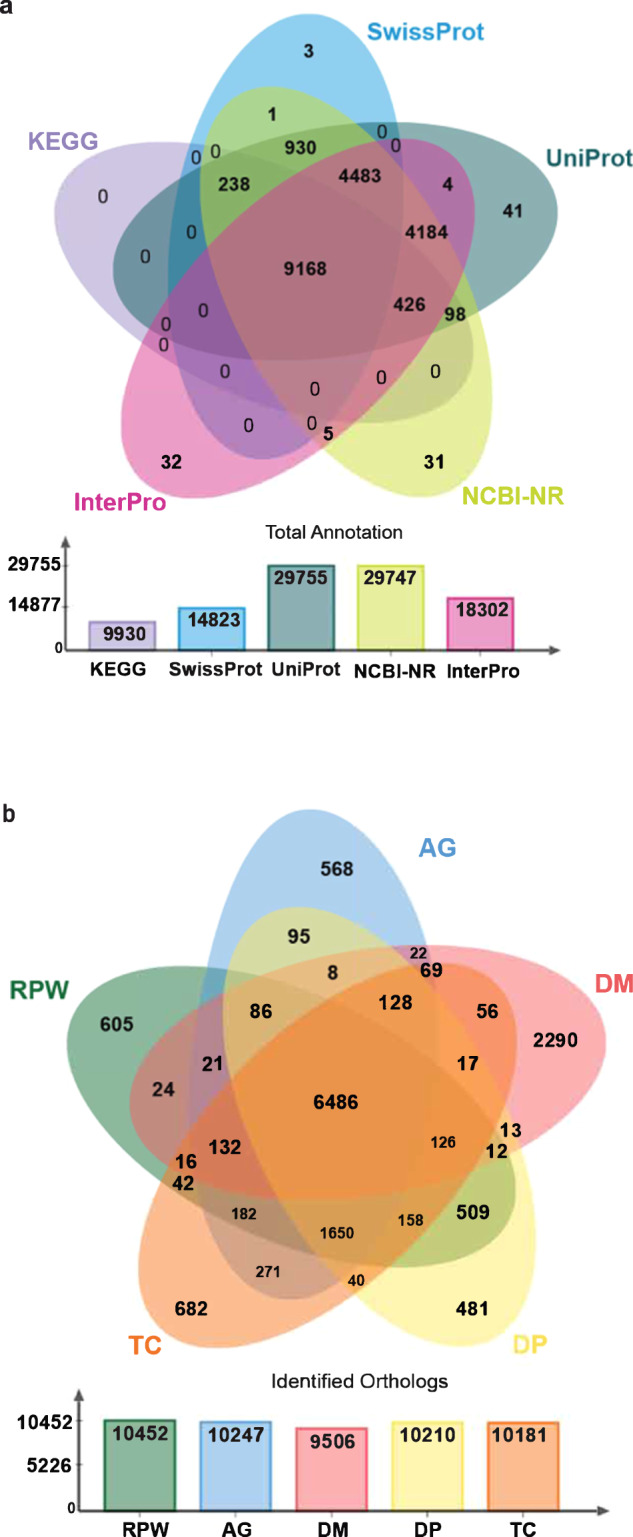
Functional annotation and orthology analysis. (**a**) Total protein annotation against KEGG, SwissProt, Uniprot, NCBI-NR and InterPro databases are show in bar graph. Venn diagram shows the shared and unique annotation with various databases. (**b**) Venn diagram shows the common and unique ortholog gene clusters in *Anoplophora glabripennis* (AG), *Drosophila melanogaster* (DM), *Dendroctonus ponderosae* (DP) and *Tribolium castaneum* (TP). Bar graph shows the total identified ortholog gene clusters in five insect species.

We carried out gene prediction on masked genome using Braker v.3 pipeline^36^. For gene annotation, we applied both *ab initio* and homology-based gene prediction approaches. In total, 226,907 insect protein sequences from 11 insect species, 20 Illumina based transcriptome and 6 PacBio based transcriptome data sets (Supplementary Table 2) were used for homology-based gene prediction. Illumina transcriptome reads were mapped against the RPW v2 genome using HiSAT2 v. 2.1 tool^37^ (-p 40 --dta–phred33 -q -S --summary-file), further using Samtools v.1.10^38^ (view -@ 40 -uhS & sort -@ 40), sorted BAM files were generated. From the BAM files transcripts were generated using StringTie v.2.1.3 program^39^. Similarly, Pacbio reads were mapped against the genome using Minimap2 v.2.17^40^ (-ax splice -t 40 -uf -C5), Samtools and SringTie and generated the sorted BAM files and transcripts files respectively. Both transcripts and protein were used for braker based gene prediction. Augustus v. 3.3.3^41^ and GeneMark-ETP v. 4.61^42^ were used for the *ab initio* gene prediction. In total, 29,666 protein coding genes were predicted from the assembled genome. Further, 1,091 tRNA and 543 rRNA genes were predicted using tRNAscan-SE v.2.0.6^43^ and Rnammer v.1.2^44^. The proteins predicted were similarity searched against NCBI-NR^45^, UniProt^18^, Swiss-Prot^46^, InterPro^47^ and KEGG^48^ databases using Blast v.2.13^49^, Diamond v.2.1.6^50^, InterPro-Scan v.5.61^51^ and KAAS^52^ tools. In total, 29,747 (~91.1%), 29,755 (~91.1%), 14823 (~45.3%), 18,302 (~56.0%) and 9,930 (~30. 4%) proteins were annotated using NR, UniProt, Swiss-Prot, InterPro and KEGG databases respectively. Among total proteins, 9,168 proteins were annotated in all databases. Finally, based on Uniprot and Interpro hits, specific Gene Ontology (GO) terms were identified for each protein (Fig. 4a).

For ortholog analysis, we retrieved proteomes of 3 Coleoptera species, *Anoplophora glabripennis* (GCF_000390285), *Dendroctonus ponderosae* (GCF_020466585), *Tribolium castaneum* (GCF_000002335) and 1 Diptera species *Drosophila melanogaster* (GCF_000001215) from NCBI-Genome database and compared with the annotated RPW protein sequences using Orthovenn2 tool^53^. In total, 15318 orthologous gene clusters were identified, among them 6,486 orthologous clusters were shared between all five insects. Overall, Coleoptera insects share most of the orthologous gene clusters (Fig. 4b). The synteny between the current genome assembly and the existing genome assembly was confirmed using D-GENIES^54^ online server by dot plot method (Supplementary Fig. 4).

## Data Records

The PacBio HiFi reads, and Omini-C reads generated during this study has been submitted in NCBI-SRA database under the BioProject id PRJNA950221^16^. The assembled whole genome of RPW (v2) is deposited in NCBI-Genome database (accession number: JASCQM000000000^15^). The predicted protein, CDS, GTF and functional annotations were deposited in Zenodo repository^55^ (10.5281/zenodo.8310271).

## Technical Validation

The isolated DNA quality and quantity were confirmed using Nanodrop (Thermo Fisher Scientific™, Waltham, MA, USA), Qubit (Thermo Fisher Scientific™, Waltham, MA, USA) and agarose gel electrophoresis method.

The quality of the RPW genome assembly was confirmed by aligning the Illumina shot-gun, PacBio HiFi and Dovetail Omini-C reads against the assembled genome followed by vertical and horizontal coverage conformation. Initially, the RPW male and female Illumina data generated from our previous study was aligned against the assembled genome using BWA program. Approximately, 99% of both male (coverage ~98% with the average depth of ~65X; Pseudochromosome coverage) and female reads aligned (coverage ~98% with the average depth of ~72X; Pseudochromosome coverage) against the RPW genome, from that ~95.2% and 94.8% of PE reads properly aligned against the genome.

We carried out BUSCO analysis (protein mode) on the predicted protein sequences and confirmed the gene prediction completeness. BUSCO analysis resulted 98.9% (1352) complete, 0.3% (4) fragmented and 0.8% (11) missing BUSCOs from the final gene prediction. Further, we aligned transcriptome reads from our previous study^11^ against the assembled genome using HiSAT tool, which resulted ~95% - ~98% of read alignment.

### Supplementary information

Supplementary Figures

Supplementary Table 1

Supplementary Table 2

## Data Availability

RepeatModeler: -database weevil_rdb -threads 100 -LTRStruct RepeatMasker: -e ncbi -pa 80 -norna -lib -a -xsmall -gff Hisat2: -p 40 --dta --phred33 -q -S --summary-file Samtools: view -@ 40 -uhS & sort -@ 40 minimap2: -ax splice -t 40 -uf -C5 rnammer: -S euk -m tsu,ssu,lsu -multi -gff tRNAscan-SE -E -o weevil_trna -f weevil_secondary -m weevil_stat -H Diamond: blastp -p 40 --query --db --evalue 1e-6 --max-hsps 1 -k 1 --outfmt 6 Interproscan: -appl pfam -dp -f TSV -goterms -iprlookup -pa -t p -cpu 90
