# Supplementary material for: Near-chromosomal-level genome of the red palm weevil (Rhynchophorus ferrugineus), a potential resource for genome-based pest control
Source: Sci Data. 2024 Jan 6;11:45. doi: 10.1038/s41597-024-02910-3 (PMC10771492; doi:10.1038/s41597-024-02910-3)
Supplement: Supplementary file 1 — Supplementary Figures [file 41597_2024_2910_MOESM1_ESM.pdf]

This document contains supplementary figures for the manuscript:

Near-chromosomal-level genome of the red palm weevil (Rhynchophorus ferrugineus), a potential resource for genome-based pest control.

Naganeeswaran Sudalaimuthuasari[1], Biduth Kundu[2], Khaled M Hazzouri[1+] and Khaled M.A. Amiri[1,2+]

1. Khalifa Center for Genetic Engineering and Biotechnology, United Arab Emirates University, Al Ain, UAE.

2. Department of Biology, College of Science, United Arab Emirates University, Al Ain, UAE.

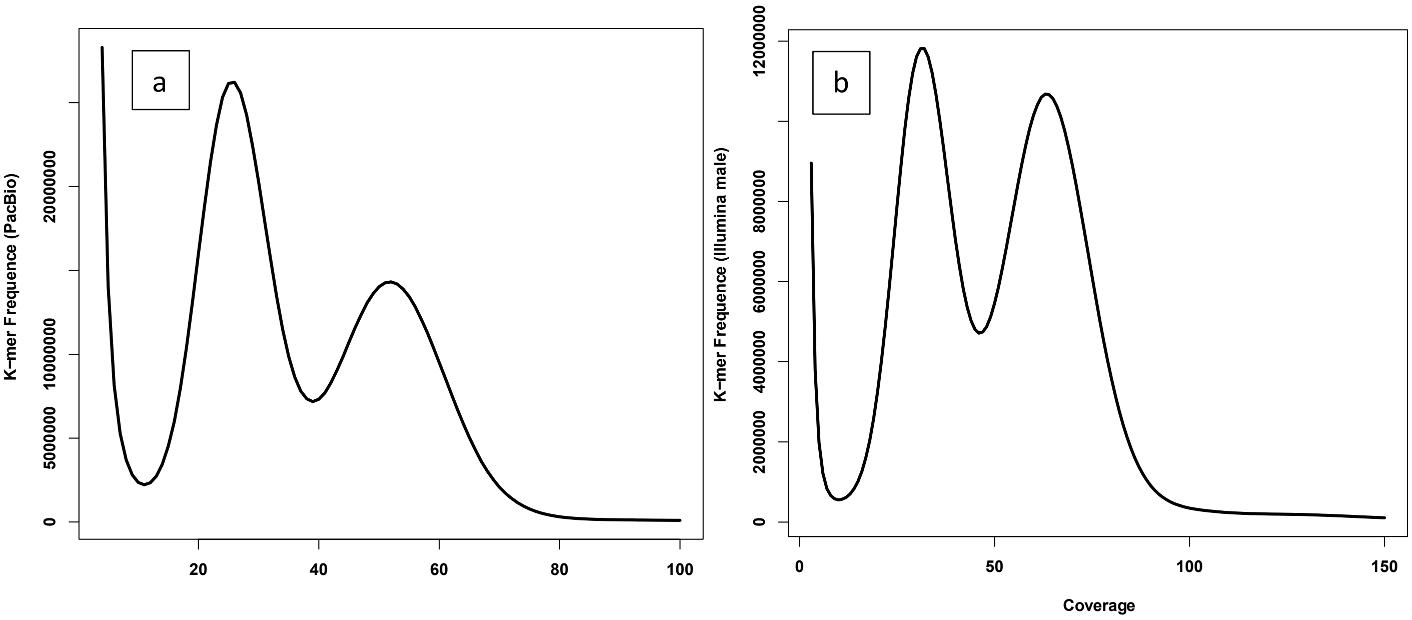

Supplementary Fig 1. K-mer distribution of PacBio HiFi long reads and Illumina shot reads: a) Figure shows the 61 bp K-mer distribution along with coverage of PacBio data. b) Figure shows the 21 bp K-mer distribution along with coverage of Illumina male data. Both Figures (1a and 1b) exhibit double peaks in the K-mer distribution, indicating the presence of a high level of heteroz-ygosity in the genome composition of the red palm weevil (RPW).

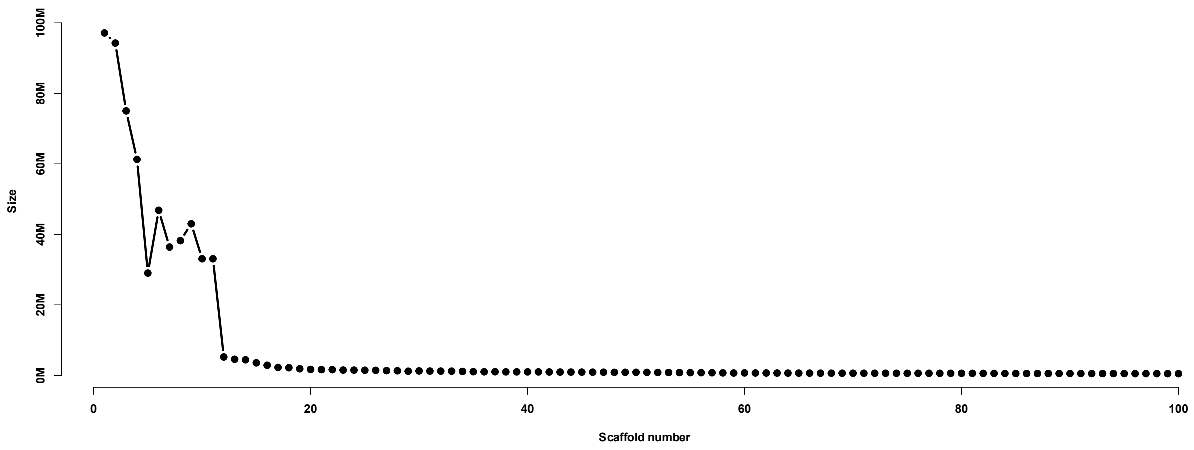

Supplementary Fig. 2 Final RPW genome assembly length distribution (Top 100 scaffolds). The first 11 scaffolds (right side) length greater than ~29 Mb, which were considered as pseudochromosomes.

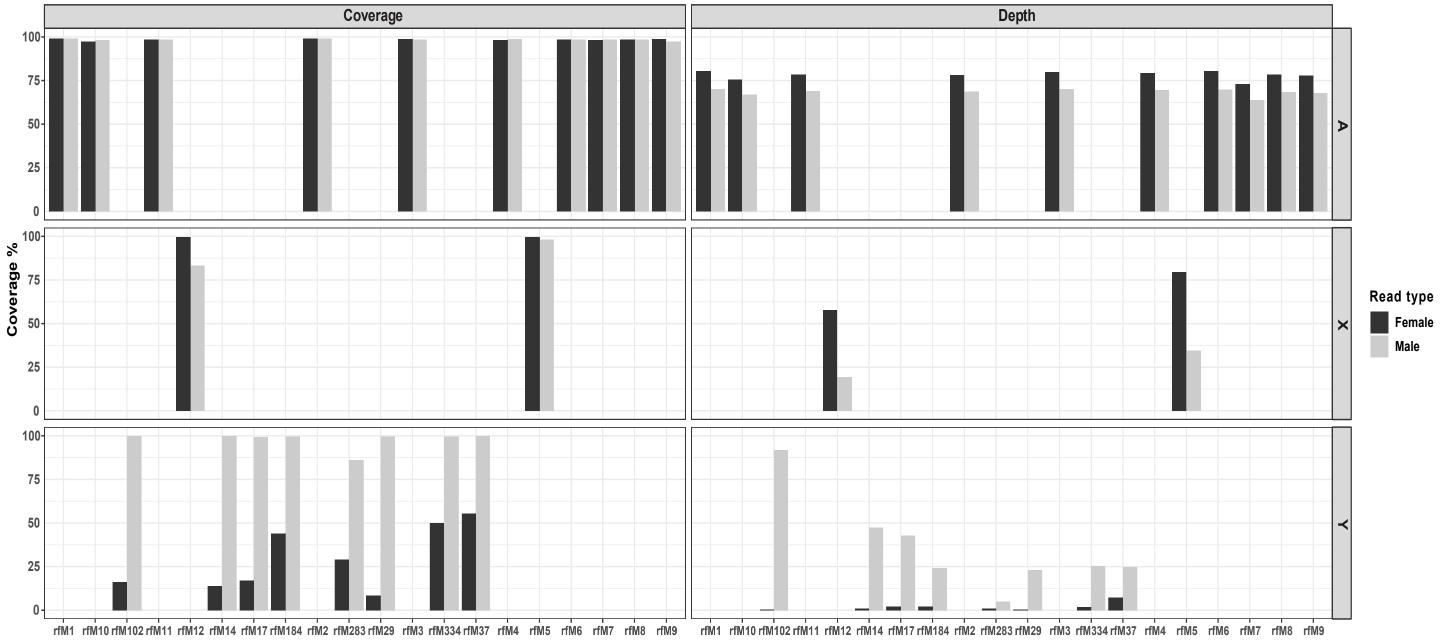

Supplementary Fig. 3 The horizontal coverage and depth (vertical coverage) distribution of autosomes (A), X-chromosome and Y- chromosome of RPW based on female and male Illumina data.

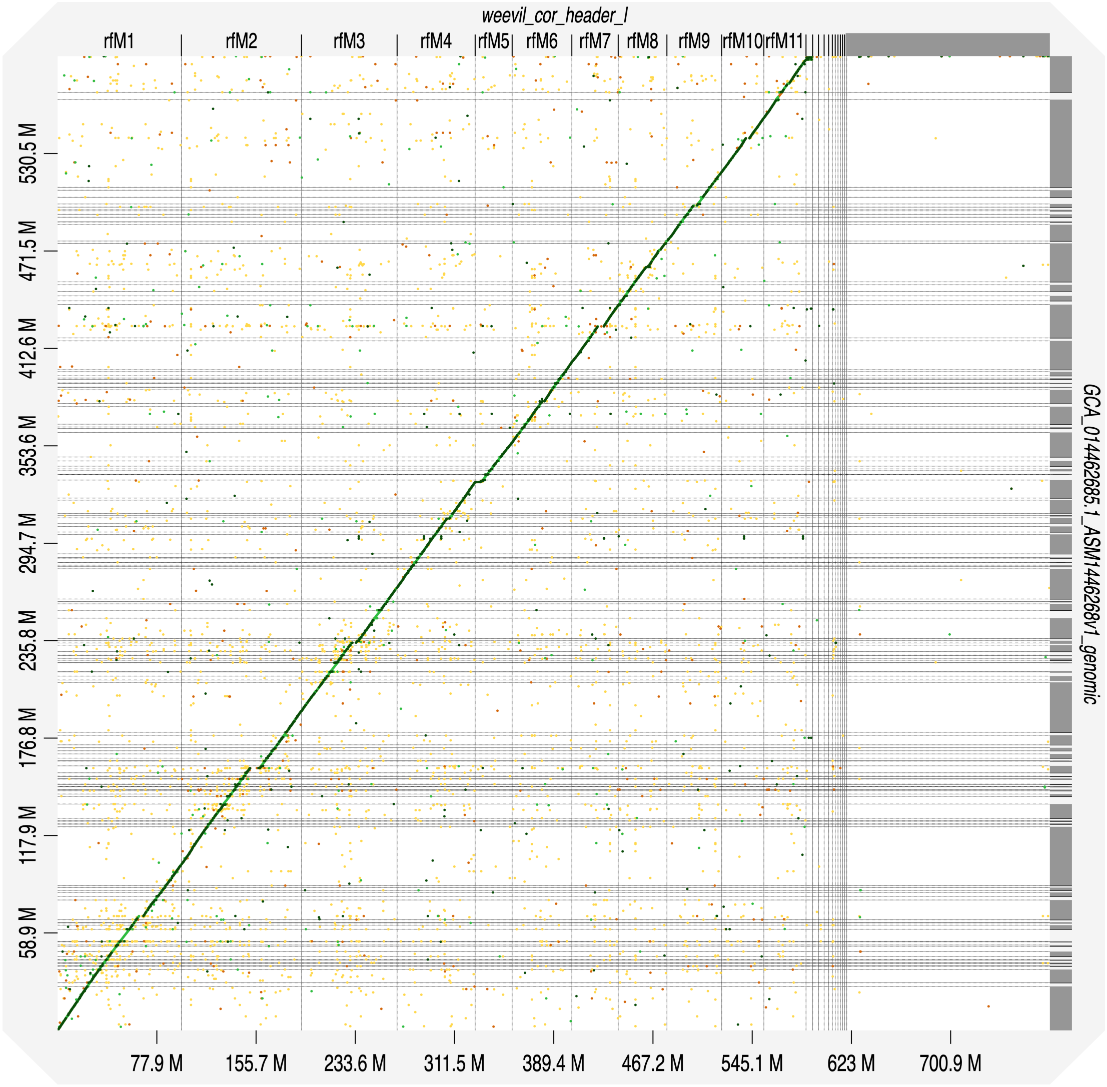

Supplementary Fig. 4 D-GENIE (https://dgenies.toulouse.inra.fr) dot plot genome comparison between RPW v2 (rfMv2) assembly with GCA_014462685 (NCBI). Diagonal green line shows high similarity between genomes.
